# Mate Choice and the Origin of Menopause

**DOI:** 10.1371/journal.pcbi.1003092

**Published:** 2013-06-13

**Authors:** Richard A. Morton, Jonathan R. Stone, Rama S. Singh

**Affiliations:** 1 Department of Biology, McMaster University, Hamilton, Ontario, Canada; 2 Origins Institute, McMaster University, Hamilton, Ontario, Canada; University of New South Wales, Australia

## Abstract

Human menopause is an unsolved evolutionary puzzle, and relationships among the factors that produced it remain understood poorly. Classic theory, involving a one-sex (female) model of human demography, suggests that genes imparting deleterious effects on post-reproductive survival will accumulate. Thus, a ‘death barrier’ should emerge beyond the maximum age for female reproduction. Under this scenario, few women would experience menopause (decreased fertility with continued survival) because few would survive much longer than they reproduced. However, no death barrier is observed in human populations. Subsequent theoretical research has shown that two-sex models, including male fertility at older ages, avoid the death barrier. Here we use a stochastic, two-sex computational model implemented by computer simulation to show how male mating preference for younger females could lead to the accumulation of mutations deleterious to female fertility and thus produce a menopausal period. Our model requires neither the initial assumption of a decline in older female fertility nor the effects of inclusive fitness through which older, non-reproducing women assist in the reproductive efforts of younger women. Our model helps to explain why such effects, observed in many societies, may be insufficient factors in elucidating the origin of menopause.

## Author Summary

The origin and evolution of menopause is understood poorly and explanations remain contentious. Virtually ignored among explanations is the effect that mate choice can exert on an evolving population. We designed and used a computational model and computer simulation to show that male mating preference for younger females in humans could have led to the accumulation of mutations deleterious to female fertility and thereby produced menopause. Our model demonstrates for the first time that neither an assumption of pre-existing diminished fertility in older women nor a requirement of benefits derived from older, non-reproducing women assisting younger women in rearing children is necessary to explain the origin of menopause.

## Introduction

Evolutionary theories of life history predict that selection should operate against living beyond the age of reproduction [1]–[3]. Postmenopausal survival is, however, a characteristic that almost is uniquely human [4], reported otherwise only in whales [5], [6] or captive chimpanzees [7]; data for other nonhuman primates [e.g.], [ 8,9] or animals [e.g., 10] are equivocal and controversial. Human life histories are exceptional in having extended dependence by juveniles and support of reproduction by older, less fertile females (i.e., through rearing). Thus, a decline of reproduction in older women constitutes an evolutionary puzzle.

Solutions may be grouped into two general explanatory categories [7], [11]–[49] (specific explanations are summarized in Table 1): 1) trade-offs between prolonged life span and reproduction; 2) fitness benefits for older, non-reproductive women through increasing the reproductive success of their offspring. Explanations in the first category are based on unique aspects of human life history, such as intelligence, social organization, and cultural transfer that allowed the evolution of longevity [45]–[51]. Female menopause (decreased fertility with continued survival) follows as a consequence of being the (assumed) ancestral state or as a trade-off favoring longevity over reproduction in women. For explanations in the second category, menopause has been considered as an adaptation that drives extended longevity beyond the decline in female fertility. Williams [3] recognized that reproduction could be extended to include individuals who promote the transmission of their own genes and that this inclusive reproduction could explain human menopause. Hawkes et al. [52] proposed that grandmothers could increase their inclusive fitness sufficiently to counteract their loss of reproduction and termed such mechanisms “grandmother effects”, although they have been recognized as being applicable to mothers as well [26]. Research in support of this hypothesis, theoretical and empirical, has focused on the possible kindred advantages of post-reproductive life and if such contributions are sufficient to explain the maintenance of menopause [2], [40], [53]–[58], as they would have to overcome the twofold cost for a grandmother to raise each grandchild rather than one of her own.

**Table 1:** Hypotheses of menopause.

Hypothesis Name	Description
**Follicular Depletion Hypothesis**	women have a fixed number of eggs, and menopause ensues when that supply becomes depleted; records of menopause in captive chimpanzees are interpreted as supporting evidence that menopause in humans results from enhanced longevity resulting in follicular depletion prior to death [7], [11]; if this interpretation were correct, then women with more children would be expected to start menopause later than would women with fewer or no children; evidence refuting this prediction may be available, as the average age at menopause appears to be very similar (58–60 years) in different societies independent of offspring number [12].
**Lifespan-artifact Hypothesis**	in the past, human longevity [13] was too short for females to experience menopause; menopause is the byproduct of an increase in longevity or life expectancy [14]–[16]; menopause may be considered as an epiphenomenon or neutral trait that may have become useful after its origin [17], [19].
**Senescence Hypothesis**	menopause is a natural effect of aging [20]; the plausibility of this hypothesis is strengthened by the assumption that reproductive aging proceeds faster than does somatic aging [21], so, unlike other physiological functions, which senesce gradually and can function at less than 100% capacity, female reproduction might have evolved as a threshold trait with minimal tolerance to perturbation.
**The Reproduction-Cost Hypothesis**	investment in reproduction is greater for women than men, leading to physiological deteriorations that amplify susceptibility to becoming infertile [22]; if this hypothesis were true, then one would expect to find a negative correlation between age at menopause and number of children, a prediction that is refuted by available data [23].
**The Mother Hypothesis**	(an adaptive version of the reproduction-cost-hypothesis) by entering into menopause, aging mothers increase the survival probability of their children [3], [24], [25]; menopause also would preclude fertilization of nonviable ova; how natural selection would select for infertility and why such a wide range of variation in the number of surviving children per family exists pose challenges to this hypothesis.
**The Grandmother Hypothesis**	menopause allows older women to contribute to the survival of their grandchildren and thus increase their inclusive fitness [25]–[37]; some authors favor a combined role of mother and grandmothers [26], [42].
**The Patriarch Hypothesis**	the origin of menopause allowed men to mate with younger women, resulting in increased longevity (for men and women) and increased status in society (for men) [43]; this hypothesis explains the extension of post-menopausal survival in women, but it does not explain why menopause evolved in the first place.
**The Absent Father Hypothesis**	reduced paternal investment and increasing maternal age were factors in the evolution of menopause [44]; this hypothesis is a complement (rather than alternative) to the grandmother hypothesis.
**Reproductive Conflict Hypothesis**	menopause is the evolutionary outcome of resource-based competition between generations (i.e., between grandmothers and their daughter-in-laws, who are unrelated and therefore ‘immigrants’ to families); on the basis of genetic relatedness between grandmothers and her daughters vs. grandchildren and between grandmothers and daughters-in-law vs. grandchildren, fitness can be optimized if daughters-in-law reproduce and grandmothers help [46]–[48].
**Evolutionary Tradeoff Hypothesis**	menopause is a tradeoff between female future production and enhanced survival of offspring [49].

Numbers in parentheses correspond to publications described in the References section.

Hamilton [1] provided the fundamental theoretical observation that genes imparting deleterious effects on post-reproductive survival will accumulate, yet human females experience menopause. More recently, researchers have recognized that Hamilton based this death barrier paradox on an inappropriate model of human demography. In his one-sex model, female [demographics] were treated as the predominant factor because they bear offspring. Male demographics were treated independently but, in fact, may differ from female demographics. Pollack [59] described a two-sex model in which a “mating preference matrix”, [M_ij_], connected male and female life histories. The mating preference matrix represented the propensity for a male of age i to form a bond with a female of age j, which might have yielded offspring if both were fertile. Such two-sex models are mathematically much more complex than are one-sex models because of the inherent non-linearity introduced by the mating preference matrix. Tuljapurkar et al. [60] showed that a two-sex model could explain life expectancy beyond the age of reproduction. In their model, older fertile males provided selection against age-dependent, mortality-causing mutations. Thus, their accumulation was prevented, and the survival of males and females was prolonged even though female fertility declined.

**Figure 1:**
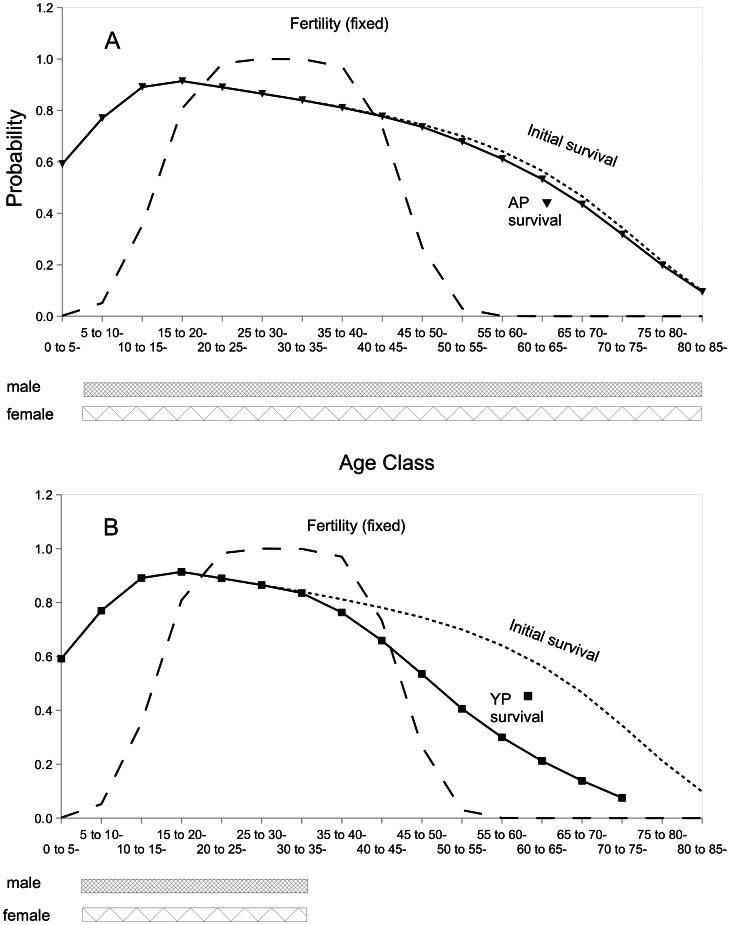
Survivorship curves for females when mortality-causing mutations are simulated. Dashed line: fixed female fertility probability. Dotted line: Gompertz-Makeham function used for initial survival probability. Solid line (with triangles in panel A and squares in panel B): Average female survival probability at simulation end. Panel A, model AP, with male and female mating allowed at all age classes after the second. Panel B, model YP, with male and female mating restricted to younger ages. Bars under the figure indicate age classes allowed to mate. Population size was held constant at 1000 males and 1000 females.

To further test the consequence of mating preference on the evolution of menopause, we modeled the effect of mutations having delayed age of onset, using stochastic, computer simulation of a population with constant size, without pre-existing diminished fertility in females, and involving mutations that affected fertility as well as mortality. Discrete age classes and time intervals were taken to correspond to five-year periods. Intrinsic fertility and survival probabilities were fixed (Table S1). Initially, only mortality-causing mutations were introduced at a constant rate into individuals in the population. Our approach differs from that adopted by Charlesworth [61], [62], who developed mathematical models to formalize the mutation accumulation hypothesis [63] that, together with the antagonistic pleiotropy hypothesis [3], [64], may be used to show how senescence can evolve by the accumulation of deleterious alleles through mutation-selection balance at frequencies that increase with their age of onset; such mutations enhance reproductive performance early in life but diminish survival late in life through physiological trade-offs. We considered mutations that affected independently mortality **and** fertility, and we established mutation-selection balance equilibria that were effected by mate choice and affected fertility. We implemented mutant alleles at any among five autosomal loci that imparted nonpleiotropic, deleterious effects (i.e., mutations that affected fertility did not affect survival and vice versa). These five loci affected survival probability for individuals in age classes beyond a particular age of onset. Individual survival thus was determined by the number and kind of mortality-causing mutations that had accumulated in the population in which a male or female were a member and, ultimately, had been inherited (details are provided in the section “Model” and tables in the Supporting Information). Eventually, simulations produced mutation-selection balance (or alternatively, fixation) in which the introduction of new mutations was balanced by the elimination, through stochastic deaths, of existing mutations. Deaths were compensated by births from pseudo-randomly chosen mating pairs. Mating pairs were formed according to an age-dependent mating preference matrix. Pairs could produce offspring only if both male and female parents were fertile, as determined from age-dependent probabilities in fertility tables. Model AP involved an age indifferent preference in the formation of mating pairs (‘All Pairs’, matrix M_ij_^AP^, Table S2), whereas model YP (‘Young Pairs’, matrix M_ij_^YP^, Table S3) involved preferences between only younger males and younger females (models used indicated by bars in Fig. 1).

## Results

Our results corroborate those obtained by Tuljapurkar et al. [60], by showing that an age indifferent preference (M_ij_^AP^) combined with extended male fertility prevents an early decline in female survival (Fig. 1A). Purifying selection (provided by male fertility) against late age of onset, mortality-causing mutations prevented them from accumulating in females, and mutation-selection balance prevented fixation (compare AP *vs.* YP in Table 2A). Plausible male and female fertility and survivorship curves were produced in equilibriums at which a menopausal decline in female fertility was established prior to decline in survival, even when a model involving preference among young pairs was used (Fig. 1B).

**Table 2:** Simulated mutant allele frequencies in females.

Locus	Age-of-Onset	Model AP	Model YP	Model YF
A: Age-dependent Mortality-Causing Mutations (sex independent)				
1	6 (25–30)	0.010 (0.001)	0.116 (0.009)	ND
2	7 (30–35)	0.018 (0.001)	1 (0)	ND
3	8 (35–40)	0.039 (0.003)	1 (0)	ND
4	9 (40–45)	0.080 (0.006)	1 (0)	ND
5	10 (45–50)	0.231 (0.022)	0.999 (0.004)	ND
B: Age-dependent, mortality-causing mutations (sex-indifferent) and sex-specific, infertility-causing mutations				
B1: Age-dependent; mortality-causing mutations				
1	6 (25–30)	0.007 (0.001)	ND	0.014 (0.001)
2	7 (30–35)	0.011 (0.001)	ND	0.024 (0.002)
3	8 (35–40)	0.023 (0.002)	ND	0.046 (0.004)
4	9 (40–45)	0.040 (0.003)	ND	0.095 (0.006)
5	10 (45–50)	0.086 (0.006)	ND	0.388 (0.032)
B2: Male-specific, infertility-causing mutations				
1	6 (25–30)	0.034 (0.002)	ND	0.033 (0.002)
2	7 (30–35)	0.049 (0.003)	ND	0.061 (0.005)
3	8 (35–40)	0.090 (0.006)	ND	0.100 (0.008)
4	9 (40–45)	0.215 (0.016)	ND	0.256 (0.024)
5	10 (45–50)	0.565 (0.036)	ND	0.730 (0.035)
B3: Female-specific, infertility-causing mutations				
1	6 (25–30)	0.034 (0.002)	ND	0.495 (0.035)
2	7 (30–35)	0.052 (0.004)	ND	0.994 (0.006)
3	8 (35–40)	0.099 (0.008)	ND	1 (0)
4	9 (40–45)	0.198 (0.018)	ND	1 (0)
5	10 (45–50)	0.684 (0.035)	ND	1 (0)

Mutant allele frequencies after 50000 time periods of computer simulation, averaged over 100 replicates. Numbers in parentheses are standard errors. Population size is 1000 females and 1000 males with two alleles at each of five loci. Polymorphisms were manifested within simulation replicates.

But this model for the evolution of menopause is not entirely satisfactory. Whereas the accumulation of age-dependent mutations that were deleterious to survival was delayed, the ancestral state already was characterized by early female loss of fertility (menopause). How did this arise? Some evidence suggests that ancestral human populations were characterized by intrinsic female menopause [55]. Human evolution, particularly through social change following brain modification, decreased extrinsic causes of mortality, and such modifications would account for survival beyond the (assumed) fixed decline in female fertility. But why did not natural selection act to extend female fertility in parallel with extended life span? And why was male fertility extended concomitantly with life span? Lahdenperä et al. [65] suggested that positive selection to increase male life span allowed reproduction throughout. This implies that female life span did not increase, because female fertility was fixed. Research foci should shift from why women live past reproductive age to why older women experience a reproductive decline.

**Figure 2:**
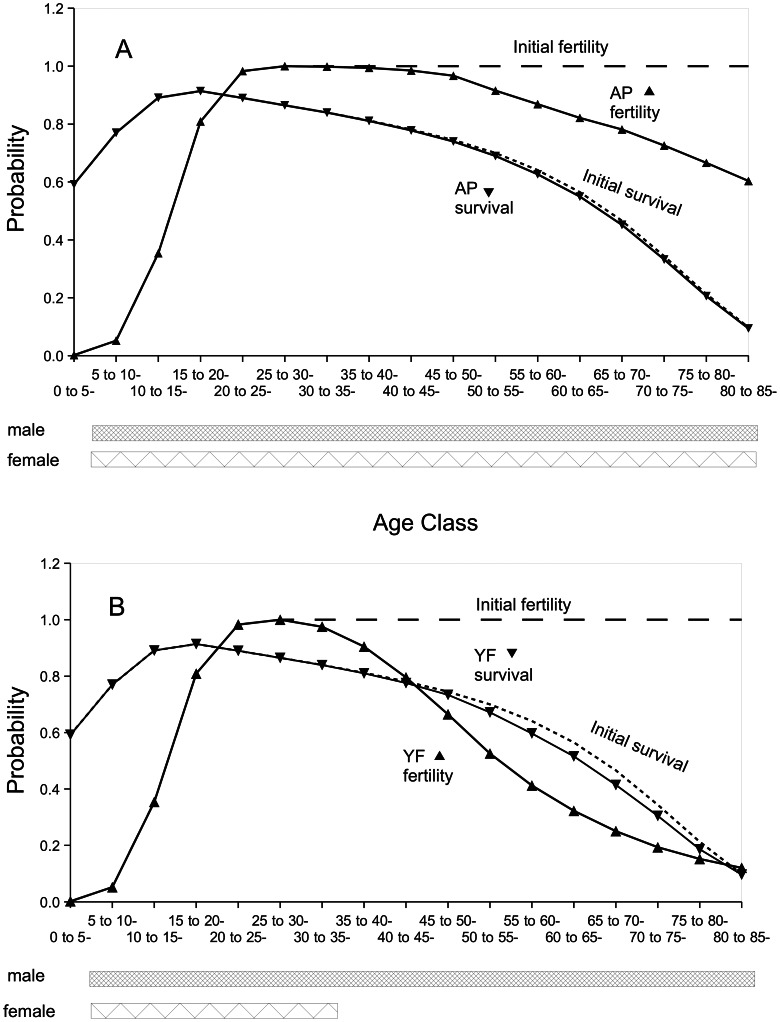
Survivorship and fertility curves for females when sex-specific, fertility-affecting mutations as well as mortality-causing mutations are simulated. Dashed line: initial female fertility probability. Dotted line: Gompertz-Makeham function used for initial survival probability. Solid line: Average female survival or fertility probability at simulation end. Panel A, Model AP – solid line, right-facing arrowheads: fertility; solid line, left-facing arrowheads: survival. Panel B, Model YF – solid line, right-facing arrowheads: fertility; solid line, left-facing arrowheads: survival. Bars under the figure indicate age classes allowed to mate. Population size was held constant at 1000 males and 1000 females.

As an alternative model for the origin and evolution of menopause, we simulated the introduction of sex-specific, infertility-causing mutations into a population for which prolonged fertility was the ancestral state for both sexes (Table S4). In this model, an early decline in female fertility was driven by a shift in male mating preference. Male behavior changed from age indifferent preference (model AP) to preference for younger females (model YF, Table S3). When an age indifferent preference matrix (M_ij_^AP^, Table S3) was used, purifying selection operated against **sex-specific**, infertility-causing mutations (Table S5), as well as **sex-indifferent**, mortality-causing mutations (Table S6). This is indicated by the equilibrium frequencies of infertility-causing and mortality-causing mutations that were obtained (Table 2 B1). Only for the locus having the oldest age of expression did infertility-causing mutations accumulate to appreciable frequencies. As infertility-causing mutations generally did not accumulate, fertility and survival remained high into old age (Fig. 2A). There was no menopause. However, with a matrix involving preference for younger females (M_ij_^YF^, Table S3), female-specific mutations with a late age of onset became nearly neutral and accumulated in the population, reaching fixation in most simulation replicates. Because male mating was retained into old age, mortality in both sexes, as well as male fertility, behaved in the same way they did with the age indifferent preference model. However, **female-specific** infertility-causing mutations accumulated (Table 2, compare model AP *vs.* YF). As a result of these mutations, female fertility declined before female survival, and female menopause arose (Fig. 2B). Male menopause never arose because male-specific infertility-causing mutations were subjected to purifying selection and did not accumulate (Table 2, B2, model YF). We emphasize that the role of the sexes would be reversed in this model if the matrix were to encode female preference for younger males (rather than male preference for younger females) and that the current role of the sexes may be interpreted equivalently as older females being out-competed completely by younger females, their dominant behavior driving the decline in female fertility. One difficulty with this model is the effect of sex-indifferent, mortality-causing mutations. These produce a sex-specific decline in survival that parallels mating preferences. This parallel decline could be avoided if mutations affecting male mortality were more frequent than were mutations affecting female mortality.

**Figure 3:**
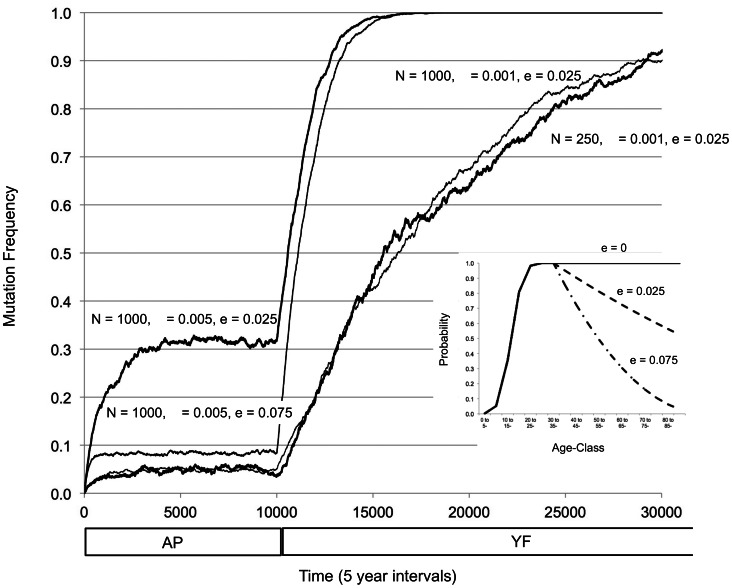
Fixation of a female-specific, infertility-causing mutation when the mating matrix is shifted from age indifferent preference model (AP) to male preference for younger females model (YF) at time period 1000. The locus is assumed to have an age of onset at age class 6 (30–35 years). Fixed population size (N), mutation rate (μ) and selection strength (e) for four different simulations (each repeated 100 times) are indicated in the figure. Y-axis is average frequency of the mutant allele in the female population. Curves: 1) N = 1000, μ = 0.005, e = 0.025; 2) N = 1000, μ = 0.005, e = 0.075; 3) N = 1000, μ = 0.001, e = 0.025; 4) N = 250, μ = 0.001, e = 0.025. The inset shows the effect of the strength of selection (e) on female fertility probability (probability if mated to produce an offspring) for a homozygous mutant female.

The difference between age indifferent preference (model AP) and preference for younger females (model YF) in producing a menopausal effect was robust across population sizes and mutation rates. We next explored the effect of shifting from a matrix encoding age indifferent preference (M^AP^; producing mutation-selection balance) to a matrix encoding preference for younger females (M^YF^; relaxation of selection leading to fixation), this time involving a single locus with age of onset in class 6 (equivalent to 30–34 years). Larger mutation rates produced more-rapid approaches to fixation when selection was reduced (Fig. 3, compare μ = 0.005 *vs.* 0.001 for N = 1000, e = 0.025). During the mutation-selection balance period (model AP), the equilibrium frequency was higher for higher mutation rates. Increasing the selection strength reduced the equilibrium frequency of mutations (Fig. 3, compare e = 0.025 *vs.* 0.075 for N = 1000, μ = 0.005) but imparted little effect on the approach to fixation during relaxed selection (YF). Decreasing the population size imparted little effect (Fig. 3, compare N = 1000 and 250 for μ = 0.001, e = 0.025). Thus, although the parameters in our model, such as age of onset, mutation rate, selection strength, and population size did affect the shift from mutation-selection balance (model AP) to relaxed selection (model YF), the shift consistently tended to produce a menopausal period.

## Discussion

Two assumptions in our model are critical to the generation of menopause. The first is a shift of male preference toward younger females. The second is the existence of female-specific mutations with detrimental effects on fertility in older women (such detrimental effects in reality may manifest physiologically as increasing Follicle Stimulating Hormone and Leutinizing Hormone levels [66] and in oocyte depletion and ultimate loss and ovulation cessation and menstruation termination [9]). Human male mating preference for younger females could be viewed as an aspect of male driven sexual selection [67], [68]. Our results demonstrate the importance of considering mating preference in population demography. Under certain conditions, mate choice can be a predominant factor in mutation accumulation and the evolution of senescence. We also have shown that it is not necessary to introduce corollary effects of inclusive fitness or grandmother effects to explain the persistence of less fertile females. That older males and females may provide reproductive benefits to others may be intuitively obvious, but these are not necessary to explain the evolutionary origin of menopause.

## Model

The computational model is encoded to simulate a population containing N males and N females evolving over time. Each individual (at time t) is assigned to an age class (1 to 18). Each individual is associated with a diploid genotype encoded at a number of loci. Mutations at a specific locus may affect either fertility or survival. Mutations may act in a sex-indifferent (i.e., either sex) or sex-specific (i.e., male or female) manner defined by an age of onset (for the locus) and with a fertility table.

Initially, individuals are assigned pseudo-randomly to age classes, without mutations. Then, a ‘burn-in’ period allows the age distribution to reach an approximate steady state.

Each time interval (representing a 5-year period) consists of determining a survival probability for each individual according to an intrinsic survival table, modified by the number and kind of mortality-affecting mutations carried. A pseudo-random number is used with the computed survival probability to determine if each individual survives and is assigned to the next age class or dies. All individuals in age class 18 die. Deaths are replaced in the population by new births assigned to the first age class.

Births are simulated by selecting a potential father pseudo-randomly from the surviving male population and a potential mother pseudo-randomly from the surviving female population. The appropriate ‘mating preference matrix’ [M_ij_] is applied to potential parents (based on their age classes i and j) to determine the probability of pair formation. Parent male and female fertility probabilities are determined using intrinsic fertility tables, modified by the number and kind of mutations carried. A pseudo-random number is used to determine if a birth occurs. If not, the birth simulation process is repeated until potential parents become actual parents.

Parents transmit their alleles to offspring according to autosomal Mendelian genetic rules; thus, if a parent is homozygous, then that parent transmits one mutation; if heterozygous, then the mutant allele is transmitted with 50% probability. At this time, the introduction of new mutations into each newborn is simulated using a pseudo-random number and the appropriate mutation rate.

Simulations were run on a Power Macintosh G5 personal computer (running OS X 10.5.8), using the C programming language (with the Apple GCC compiler). Computational model code is accessible online in the Supporting Information (Text S1).

## Supporting Information

Table S1 **Intrinsic fertility and survival probabilities.**
(DOC)

Table S2 **Mating preference matrix M^AP^.**
(XLS)

Table S3 **Mating preference matrix M^YP^.**
(XLS)

Table S4 **Intrinsic fertility (prolonged) and survival.**
(DOC)

Table S5 **Female-specific, infertility-causing loci.**
(DOC)

Table S6 **Sex-indifferent, mortality-causing mutations.**
(DOC)

Text S1 **Computational model code.**
(TXT)
