# Supplementary material for: Mate Choice and the Origin of Menopause
Source: PLoS Comput Biol. 2013 Jun 13;9(6):e1003092. doi: 10.1371/journal.pcbi.1003092 (PMC3681637; doi:10.1371/journal.pcbi.1003092)
Supplement: Table S4 — Intrinsic fertility (prolonged) and survival. (DOC) [file pcbi.1003092.s004.doc]

**Table S4. Intrinsic fertility (prolonged) and survival.**

| **Age Class** | **Age range (years)** | **Fertility** | **Survival** |
| --- | --- | --- | --- |
| 1 | 0 to 5- | 0.002 | 0.5920 |
| 2 | 5 to 10- | 0.052 | 0.7700 |
| 3 | 10 to 15- | 0.354 | 0.8910 |
| 4 | 15 to 20- | 0.809 | 0.9140 |
| 5 | 20 to 25- | 0.983 | 0.8900 |
| 6 | 25 to 30- | 1.000 | 0.8650 |
| 7 | 30 to 35- | 1.000 | 0.8400 |
| 8 | 35 to 40- | 1.000 | 0.8120 |
| 9 | 40 to 45- | 1.000 | 0.7810 |
| 10 | 45 to 50- | 1.000 | 0.7450 |
| 11 | 50 to 55- | 1.000 | 0.7000 |
| 12 | 55 to 60- | 1.000 | 0.6410 |
| 13 | 60 to 65- | 1.000 | 0.5650 |
| 14 | 65 to 70- | 1.000 | 0.4660 |
| 15 | 70 to 75- | 1.000 | 0.3440 |
| 16 | 75 to 80- | 1.000 | 0.2130 |
| 17 | 80 to 85- | 1.000 | 0.0980 |
| 18 | 85 to 90- | 1.000 | 0.0000 |
