# Supplementary material for: Mate Choice and the Origin of Menopause
Source: PLoS Comput Biol. 2013 Jun 13;9(6):e1003092. doi: 10.1371/journal.pcbi.1003092 (PMC3681637; doi:10.1371/journal.pcbi.1003092)
Supplement: Table S5 — Female-specific, infertility-causing loci. (DOC) [file pcbi.1003092.s005.doc]

**Table S5. Female-specific, infertility-causing loci.**

| **Locus** | **Age Class, age-of-onset** | **Age Range (years), age-of-onset** |
| --- | --- | --- |
| 1 | 6 | 25-30 |
| 2 | 7 | 30-35 |
| 3 | 8 | 35-40 |
| 4 | 9 | 40-45 |
| 5 | 10 | 45-50 |
